# Physical characterization of frozen fruits from eight cultivars of the North American pawpaw (*Asimina triloba*)

**DOI:** 10.3389/fnut.2022.936192

**Published:** 2022-10-18

**Authors:** Bezalel Adainoo, Brendan Crowell, Andrew L. Thomas, Chung-Ho Lin, Zhen Cai, Patrick Byers, Michael Gold, Kiruba Krishnaswamy

**Affiliations:** ^1^Division of Food Systems and Bioengineering, University of Missouri, Columbia, MO, United States; ^2^Department of Biomedical, Biological and Chemical Engineering, University of Missouri, Columbia, MO, United States; ^3^Division of Plant Science and Technology, Southwest Research Center, University of Missouri, Mt. Vernon, MO, United States; ^4^School of Natural Resources, Centre for Agroforestry, University of Missouri, Columbia, MO, United States; ^5^University of Missouri Extension Center, Marshfield, MO, United States

**Keywords:** *Asimin triloba*, Annonaceae, pawpaw, pawpaw processing, tropical fruit

## Abstract

Pawpaw (*Asimina triloba* [L.] Dunal) is an underutilized fruit native to North America. The fruit has a short shelf life, and browns and softens rapidly after harvesting. These characteristics present a challenge to the advancement of pawpaw as an economically viable specialty crop. This study evaluated the physical characteristics of frozen fruits from eight cultivars of the pawpaw fruit to establish the processing potential of pawpaw fruits. The results show that freeze-thaw cycle may have influenced the peel thickness, peel color, and pulp color of the fruits. Fruits of the Susquehanna cultivar had the highest fruit weight and pulp weight, making them potentially the most suitable for pulp processing. The pawpaw fruits had almost neutral pH ranging between 6.07 ± 0.21 and 6.47 ± 0.11, which could contribute to the rapid browning on exposure to air since an acidic pH is important for slowing enzymatic browning. To aid pawpaw juice extraction, enzymatic treatments may be necessary to increase the juice yield from the pulp. Overleese fruits may be the best for pawpaw juice production. These findings can aid in the selection of processing equipment and guide processors in their efforts to utilize pawpaw fruits to avoid postharvest and post-processing losses.

## Highlights

- The physical characteristics of frozen fruits from eight cultivars of North American pawpaw were investigated.
- The freeze-thaw cycle may have influenced the peel thickness, peel color, and pulp color of the fruits from the different cultivars differently.
- The fruits had almost neutral pH which could explain the rapid browning of the pulp since an acidic pH is important for slowing enzymatic browning.
- There is potential for the use of pawpaw pulp in the production of juices, jams, jellies, and several other value-added products.

## Introduction

The North American pawpaw (*Asimina triloba* [L.] Dunal) is a fruit tree that belongs to the same family (Annonaceae) as several widely cultivated tropical fruit trees such as soursop/graviola (*Annona muricata* L.), custard apple (*Annona reticulata* L.) and sugar apple (*Annona squamosa* L.) (1). The pawpaw is one of the few temperate species of this family and is native to the Eastern region of North America. Pawpaw grows best in places that experience hot summers and cold winters (2). Its distribution spans from the west of New York to southwestern Ontario southwards through Michigan, Illinois, Missouri, and further south to eastern Texas and Florida. The fruit is currently being grown in other countries including South Korea, Japan, Italy, China, Israel, Romania, Portugal, Nigeria, and Belgium (1, 3, 4).

Between 1900 and 1960, more than 56 pawpaw cultivars were named, however, with time, some of them were lost as they were no longer cultivated (5). Presently, there are about 47 known pawpaw cultivars, and these include both wild selections and bred cultivars (6). Pawpaw fruits are mostly asymmetrical, having an oblong-cylindrical shape with some having globular or arched shapes (7). Fruits of the various cultivars differ in their rate of growth and ripening, and physical characteristics such as fruit size, color, texture, and percent of seeds (6). Although the flavor of the fruit has commonly been described to be similar to the combination of banana, mango, and pineapple flavors, other flavor descriptors such as apple, melon, and fresh flavors have been used by trained sensory panelists to describe the flavor of specific cultivars of pawpaw fruit (8, 9). The fruit's unique flavor and aroma make it suitable for potential applications in baby foods, fruit drinks, ice cream, and as a substitute for bananas in various foods (10). The fruit contains 79.14% moisture, 0.38% ash, 1.51% protein, 0.36% lipid, 2.47% crude fiber, 18.61% carbohydrates, and 3.03% dietary fiber (3). Further, it is known to be a good source of β-carotene, polyphenols, antioxidants, and other important compounds (3, 11).

Pawpaw fruits can weigh up to 1 kg (1, 12). Some of the cultivars that yield large fruits include Convis, IXL, Lynn's Favorite, Overleese, SAA-Overleese, Shenandoah, and Susquehanna, whereas those that yield small fruits are LA Native, Middletown, Rappahannock, and Wilson (6).

It has been observed that a change in the intensity of the peel's green color is not a good measure of the ripeness of the pawpaw fruit because this color change is not consistent for all the genotypes (13). However, the peels change color from green to yellow to brownish black as ripening progresses and the pulp color of a ripe pawpaw fruit ranges from creamy white to yellow to orange (1). Additionally, the pulp browns when exposed to air. This color change is caused by the action of polyphenol oxidases in the fruit pulp (14). The fruit contains two rows of black seeds that are about the size of almonds.

Pawpaw fruits have a short shelf life. As pawpaw fruits ripen, the soluble solids concentration increases, however, this is not a good indicator of ripeness (1). The fruits soften within 3 days after harvesting due to their high ethylene production and climacteric respiration (13). By day 5 after harvesting (without refrigeration), the fruit often becomes overripe and too soft to handle (15). These factors coupled with the rapid color changes that occur in the peel and pulp make processing the pawpaw fruit a challenge.

This study aimed to assess the physical characteristics of the frozen pawpaw fruits from eight different cultivars to establish a basis for their processing potential. Understanding the physical characteristics of the pawpaw fruit is important for the selection of advanced cultivars, as well as the design of appropriate processing equipment, to allow for the industrial processing of the fruit and ensure there are no significant losses during processing operations.

## Materials and methods

### Pawpaw and mango samples

Fifty-three (53) pawpaw fruits from eight cultivars (10–35, PA-Golden, Shenandoah, Sunflower, Susquehanna, Wells, Overleese, and Wilson) were harvested from two orchards (designated Lower and Upper orchards; 1 km apart) at the Southwest Research Center of the University of Missouri, Mt. Vernon, MO (lat. 37.08582, long. −93.86713, and lat. 37.07146, long. −93.87870 respectively). The Lower orchard had a fertile alluvial soil that was deep and well-drained, whereas the Upper orchard had a less fertile soil with a shallow fragipan that required more irrigation than the Lower orchard. The trees generally grew larger and more vigorously in the Lower Orchard. The fruits were harvested at peak ripeness (determined by the pitting on the skin when the fruit is gently pressed with a finger) in Sept./Oct. 2020. The fruit from the respective cultivars were separated by placing them in separate zippered plastic storage bags and stored whole in a deep freezer (−18°C) immediately after harvesting for 14 days prior to transportation to the laboratory for analyses. Fruits were thawed in tepid water at ~25°C for approximately 10 min before analyses. Mango fruits purchased from Walmart in Columbia, Missouri were used as control.

### Fruit and fruit component weight and size

**Figure 1:**
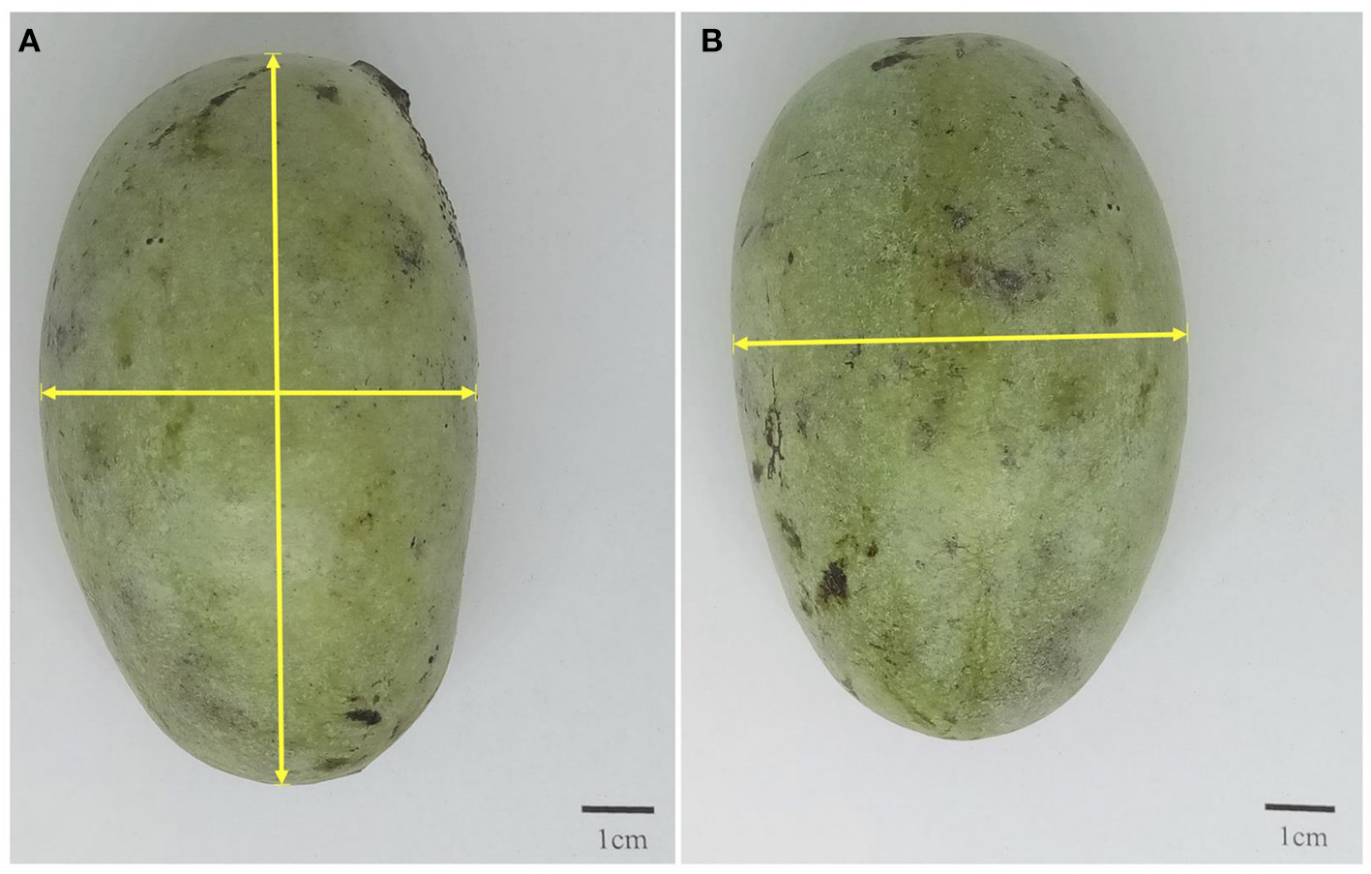
Pictorial demonstration of how **(A)** fruit length and width and **(B)** thickness were measured.

The total weight, seed weight, and peel weight for each fruit were measured using an analytical balance. Pulp weight was obtained by difference. The number of seeds per fruit were recorded, except for the mango fruits, which only have one seed. Peel thickness was measured with a Vernier caliper (16). The fruit length, width, and thickness were also measured with a Vernier caliper as demonstrated in Figures 1A,B. All measurements were done in five replicates.

### Fruit color

Fruit peel color, outside pulp color (the pulp just beneath the peel), internal pulp color, and seed color were measured according to the method described by Nambi et al. (17) using the Hunter LAB color meter (Chroma Meter CR-410, Konica Minolta). All color readings were done in five replicates at five different points on the peel, outside pulp, internal pulp, and seeds. The L^*^, a^*^, and b^*^ readings were recorded where, L^*^ is the degree of lightness to darkness, a^*^ is the degree of redness to greenness, and b^*^ is the degree of yellowness to blueness.

### Fruit shape index

Fruit shape index (FSI) was measured as the ratio of the maximum fruit length to the maximum fruit width as described by Brewer et al. (18).


$$\text{Fruit shape index}=\;\frac{\text{Maximum fruit length}}{\text{Maximum fruit width}}$$

### Fruit volume

Fruit volume was measured by the displacement method using a graduated measuring cylinder (19). The measuring cylinder was filled with water to a specific volume and the change in displacement of water after gently dropping fruit into the water was recorded as the volume of the fruit in cm^3^. The measurements were taken in five replicates for each fruit.

### Pulp density

The density of pawpaw and mango pulps was determined according to the procedure described by Bon et al. (20) with some modifications. A pycnometer (Ultrapycnometer 1000, Quantachrome Instruments) and an analytical balance were used to determine the pulp density in triplicates at 25°C. 5 g of pulp was first weighed into the small pycnometer cell. The pycnometer was set to take five density readings and take averages of the five readings. This was done in triplicates.

### Determination of juice content

The juice content was determined according to the methods described by Agbaje et al. (21) and Jamil et al. (22). Fruits were washed with tap water followed by distilled water to remove foreign materials from the fruit. The fruit was hand peeled and the pulp separated from the peels and seeds, then blended to reduce size and aid juice extraction. The juice in a known weight of the blended fruit pulp was extracted using a clean white muslin cloth. The juice content was calculated as a percentage of the weight of the fruit. Juice contents were determined in triplicates.


$$\text{Juice content }=\;\frac{\text{Weight of extracted juice}}{\text{Weight of blended fruit}}\;\times \text{100}$$

### Determination of pH and titratable acidity

The pH of the fruit was measured using a digital pH meter (SevenCompact S220, Mettler Toledo) at room temperature (25°C). The measurements were taken in five replicates. Titratable acidity was determined according to the AOAC Official Method 942.15 (23). Since the pulp of the fruits was quite dry at the time of the titratable acidity experiment, 5 g of the fruit pulp was mixed with 25 g of distilled water, blended in a kitchen blender for 2 min to obtain a homogeneous mixture, and titrated against 0.1 N NaOH using phenolphthalein as indicator. The analyses were performed in triplicates and reported as acetic acid equivalents since the predominant acid in pawpaw is acetic acid (3).


$$\text{Titratable acidity =}\frac{\text{NaOH normality }\times \text{ Titre value }\times \text{ Acetic acid eq}.\text{ weight }\times \text{100}}{\text{Sample weight }\times \text{1000}}$$

Acetic acid eq. weight = 60.052 g

### Determination of total soluble solids

Total soluble solids (TSS) content was measured according to the AOAC Official Method 932.14 C (23) using a digital refractometer (HI96800, Hanna Instruments) at room temperature (~25°C). 5g of the fruit pulp was mixed with 25 ml of distilled water and blended in a kitchen blender for 2 min to obtain a homogeneous mixture. The readings were multiplied by the dilution factor (5). The total soluble solids measurements were taken in triplicates and recorded as °Brix.

### Determination of thermophysical properties

The thermophysical properties of pawpaw pulp (only Sunflower cultivar) was determined by differential scanning calorimetry (DSC) as described by Gundurao et al. (24) with some modifications. The differential scanning calorimeter (TA Q20, TA Instruments) calibrated with indium for heat flow and the temperature was equipped with a cooling system that monitored temperatures down to −90°C. Nitrogen gas was used as a purge gas with a flow rate of 50 ml/min. About 14 mg of pawpaw pulp was weighed into aluminum pans which were hermetically sealed to avoid moisture loss. An empty sealed aluminum pan was used as a reference. To determine the glass transition temperature and the change in specific heat capacity, sealed pans with pawpaw pulp samples were cooled to −30°C and subjected to a programmed heating rate of 10°C/min to 200°C. The DSC data were analyzed with the Universal Analysis Software (version 4.5A) for thermal analysis.

### Microstructure of pawpaw pulp (scanning electron microscopy)

Pulp samples from near the seeds and pulp samples further from the seeds were collected from Susquehanna pawpaw fruits and processed for scanning electron microscopy (SEM). Unless otherwise stated, all reagents were purchased from Electron Microscopy Sciences and all specimen preparation was performed at the Electron Microscopy Core Facility, University of Missouri. Tissues were fixed in 2% paraformaldehyde, and 2% glutaraldehyde in 100 mM sodium cacodylate buffer pH = 7.35. Next, fixed tissues were rinsed with 100 mM sodium cacodylate buffer, pH 7.35 containing 130 mM sucrose. Secondary fixation was performed using 1% osmium tetroxide (Ted Pella, Inc., California, USA) in cacodylate buffer using a Pelco Biowave (Ted Pella, Inc., California, USA) operated at 100 W for 1 min. Specimens were next incubated at 4°C for 1 h, then rinsed with cacodylate buffer and further with distilled water. Using the Pelco Biowave, a graded dehydration series (per exchange, 100 Watts for 40 s) was performed using ethanol. Samples were dried using the Tousimis Autosamdri 815 (Tousimis, Maryland, USA) and samples were sputter coated with 20 nm of platinum using the EMS 150T-ES. Sputter Coater Images were acquired with a FEI Quanta 600F scanning electron microscope (FEI, Oregon, USA) at a voltage of 5.00 kV and magnifications of 100x, 500x, and 1,000x.

### Statistical analysis

All experimental data are presented as mean values ± SD (standard deviation). The data were analyzed by analysis of variance (ANOVA) and Tukey's test (*p* < 0.05) for significant differences using JMP 14.0.0 software (SAS Institute Inc., Cary, USA). Microsoft Excel version 16.46 was used for further processing of the data into tables and graphs.

## Results

### Size and morphological characteristics of pawpaw

**Figure 2:**
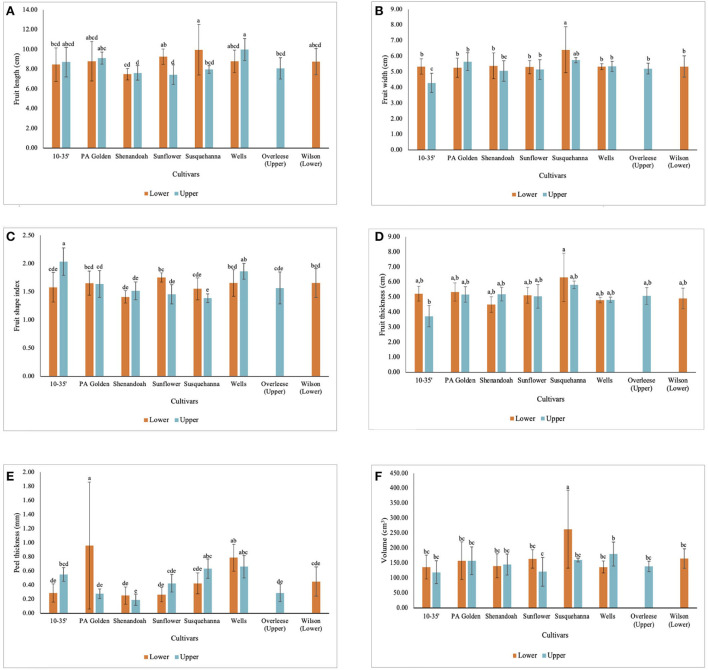
Dimensional characteristics of pawpaw fruits from different cultivars in the Lower and Upper orchards showing **(A)** fruit length, **(B)** fruit width, **(C)** fruit shape index, **(D)** fruit thickness, **(E)** peel thickness and **(F)** volume of fruits.

The fruit size data shows that though the Lower orchard had better soil conditions than the Upper orchard, the differences in the soil, and environmental conditions in which pawpaw fruits are grown, affect the fruit size of the cultivars differently. Fruits of the 10–35, PA-Golden, Shenandoah, and Wells cultivars from the Upper orchard had slightly higher average lengths than fruits of those cultivars in the Lower orchard. However, there were no statistical differences between fruits of these cultivars from the two orchards at *p* < 0.05 as shown in Figure 2A. Meanwhile, fruits of the Sunflower and Susquehanna cultivars in the Lower orchard were longer than the fruits of these cultivars in the Upper orchard with statistically significant differences at *p* < 0.05. Further, apart from the statistical differences between the widths of the 10–35 fruits in the Lower orchard and the Upper orchard, there were no significant differences between the widths of the fruits from the two orchards for all the other cultivars except for the Susquehanna fruits from the Lower orchard which had a significantly higher fruit width (Figure 2B).

From the fruit shape index (FSI) data (Table 1 and Figure 2C), fruits of the 10–35 cultivar in the Upper orchard had significantly higher FSI (2.04 ± 0.24) than those in the Lower orchard and all the other cultivars. The differences in the fruit thickness among the cultivars were statistically insignificant at *p* < 0.05 (Figure 2D). From the data obtained, Susquehanna fruits recorded the highest average volume (217 ± 110 cm^3^), which was also significantly different from the fruits from the other cultivars at *p* < 0.0001 (Table 1). Further, among the fruits from the two orchards, Wells and Sunflower fruits harvested from the Upper orchard and the Susquehanna fruits from the Lower orchard showed significant differences in volume. The volume of the fruits from the other cultivars were not statistically different at *p* < 0.05 (Figure 2F).

**Table 1:** Morphological characteristics of pawpaw fruits from different cultivars grown in southwest Missouri, 2020.

**Cultivar**	**Volume (cm^3^)**	**Fruit length (cm)**	**Fruit width (cm)**	**Fruit thickness (cm)**	**Fruit shape index**	**Peel thickness (mm)**	**Fruit weight (g)**	**Pulp weight (g)**	**Peel weight (g)**	**Seed weight (g)**	**Number of seeds**
10–35	130 ± 40^b^	8.56 ± 1.62^abc^	4.95 ± 0.74^c^	4.64 ± 0.94^a^	1.75 ± 0.34^a^	0.38 ± 0.18^bcd^	137 ± 44^b^	122 ± 37^b^	10.1 ± 4.5^d^	6.4 ± 2.8^d^	4–8
Overleese	139 ± 17^b^	8.07 ± 1.09^bc^	5.20 ± 0.35^bc^	5.08 ± 0.56^a^	1.57 ± 0.28^ab^	0.29 ± 0.12^cd^	143 ± 14^b^	109 ± 10^b^	24.9 ± 2.9^ab^	8.6 ± 1.7^bcd^	4–13
PA Golden	158 ± 54^b^	8.99 ± 1.40^ab^	5.48 ± 0.62^b^	5.25 ± 0.56^a^	1.64 ± 0.23^ab^	0.57 ± 0.68^ab^	171 ± 50^b^	143 ± 39^b^	17.1 ± 14.7^bcd^	10.5 ± 2.8^bc^	3–12
Shenandoah	143 ± 37^b^	7.58 ± 0.75^c^	5.16 ± 0.66^bc^	4.96 ± 0.60^a^	1.48 ± 0.15^b^	0.21 ± 0.10^d^	143 ± 38^b^	117 ± 35^b^	16.7 ± 2.3^bcd^	9.2 ± 2.5^bcd^	4–8
Sunflower	142 ± 46^b^	8.36 ± 1.27^bc^	5.23 ± 0.54^bc^	5.09 ± 0.67^a^	1.60 ± 0.20^ab^	0.34 ± 0.14^cd^	148 ± 47^b^	123 ± 39^b^	14.6 ± 7.8^cd^	11.1 ± 5.2^b^	4–12
Susquehanna	217 ± 110^a^	9.07 ± 2.16^ab^	6.11 ± 1.15^a^	6.09 ± 1.23^a^	1.48 ± 0.17^b^	0.51 ± 0.18^abc^	241 ± 135^a^	208 ± 118^a^	24.7 ± 16.0^a^	8.2 ± 4.7^cd^	3–13
Wells	160 ± 39^b^	9.41 ± 1.27^a^	5.34 ± 0.27^bc^	4.81 ± 0.18^a^	1.76 ± 0.22^a^	0.72 ± 0.18^a^	160 ± 37^b^	125 ± 25^b^	18.0 ± 8.9^abc^	17.2 ± 3.9^a^	5–13
Wilson	165 ± 33^b^	8.77 ± 1.33^abc^	5.34 ± 0.68^bc^	4.92 ± 0.68^a^	1.66 ± 0.26^ab^	0.45 ± 0.21^bcd^	167 ± 29^b^	138 ± 22^b^	12.1 ± 5.2^cd^	16.6 ± 2.5^a^	8–10
Control (Mango Fruit)	324 ± 3	9.85 ± 0.18	7.89 ± 0.19	7.20 ± 0.20	1.25 ± 0.05	2.76 ± 0.56	334 ± 20	201 ± 20	97.1 ± 14.2	35.4 ± 3.7	-

Values with the same superscripts are statistically similar at p < 0.05.

Fruit shape index is the ratio of the maximum fruit length to the maximum fruit width.

**Figure 3:**
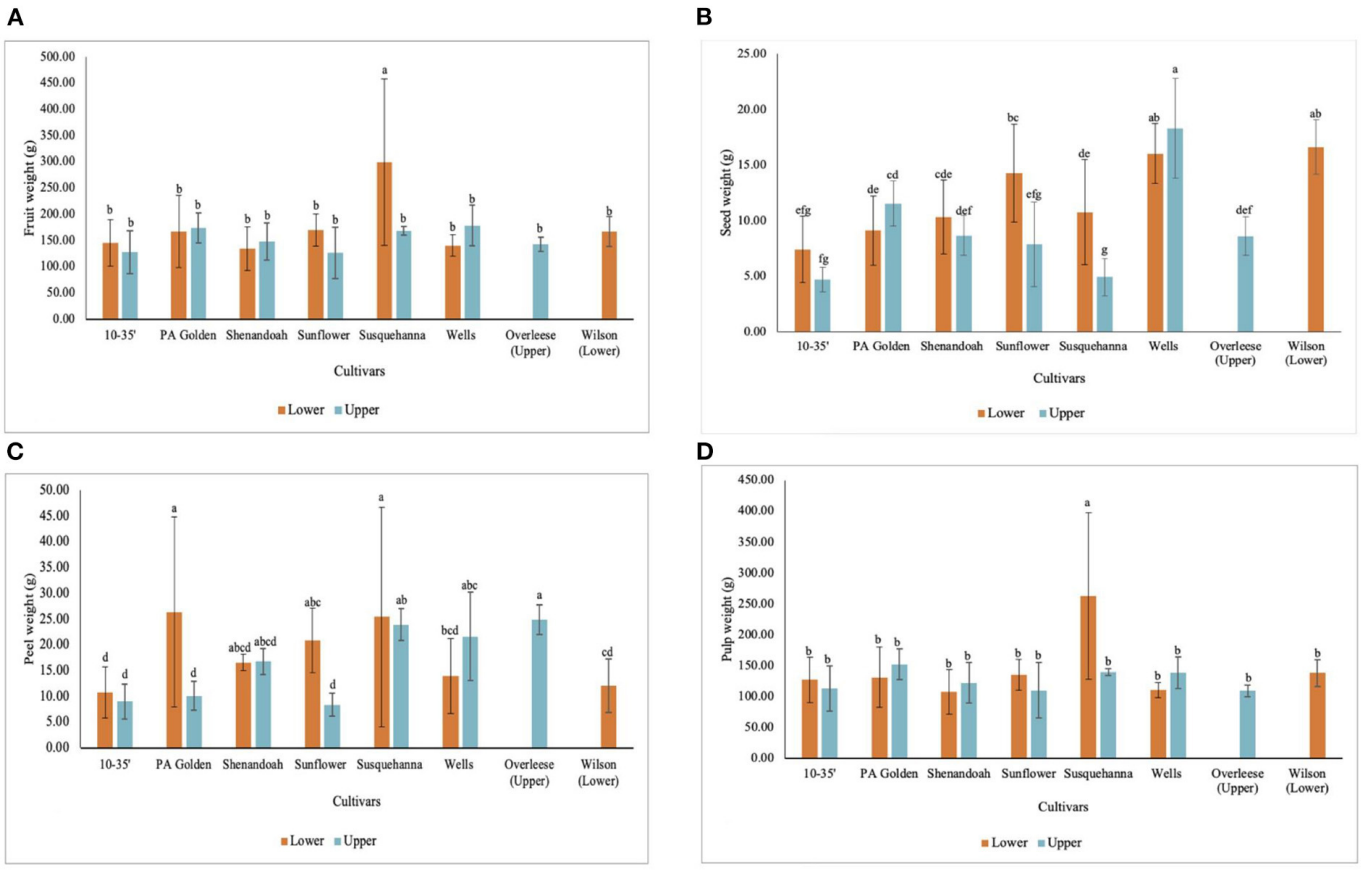
Weight of **(A)** fruits, **(B)** seeds, **(C)** peels, and **(D)** pulp of pawpaw fruits from different cultivars in the Lower and Upper orchards.

The environmental and soil differences between the Lower and Upper orchards did not affect the weights of the fruits except for the Susquehanna fruits from the Lower orchard, which had significantly heavier fruits (299 ± 158 g) compared to the other cultivars studied (Figure 3A). There was no statistical difference (*p* < 0.05) in the weights of the fruits among the other cultivars studied (Table 1 and Figure 3A). Also, the number of seeds per fruit varied widely among the cultivars. Some PA Golden and Susquehanna fruits had as few as three seeds per fruit, whilst some Overleese, Susquehanna, and Wells fruits had as many as 13 seeds per fruit. This contributed to the wide variations in the seed weights as shown in Figure 3B. On average, seeds in the Lower orchard 10–35, Shenandoah, Sunflower, and Susquehanna fruits weighed more than seeds of the same cultivars in the Upper orchard (Figure 3B). Similarly, there were wide variations in the peel weights (Table 1 and Figure 3C).

Some of the pawpaw fruits studied had thin peels while others had thick peels because layers of the outside pulp firmly adhered to the peels. However, this was not consistent for all the fruits, which explains why the peel thickness of the Susquehanna fruits from the Lower orchard was significantly lower than that of the PA-Golden fruits from the Lower orchard (Figure 2E) but the peel weights of both Susquehanna and PA-Golden fruits from the Lower orchard were not significantly different (Figure 3C).

Susquehanna fruits recorded the highest pulp weight (208 ± 118 g). The data (Table 1 and Figure 3D) show that there were no significant differences (*p* < 0.05) in the pulp weights of the other cultivars tested, although PA-Golden and Wilson fruits had slightly more pulp than the others. Moreover, the better soil conditions of the Lower orchard favored the pulp weight of the Susquehanna, 10–35, and Sunflower cultivars, but not the PA Golden, Shenandoah, and Wells cultivars.

### Pawpaw color

There were statistical differences in peel color among fruits of the same cultivar from the different orchards (*p* < 0.0001) and among fruits of different cultivars (*p* < 0.0001). The peels of the Sunflower cultivar fruits were the lightest (43.9 ± 3.6 L^*^) and had the highest yellowness (24.6 ± 6.2 b^*^) among the cultivars studied, whereas the peels of the Wells cultivar fruits were the darkest (34.6 ± 5.1 L^*^) of the cultivars studied (Table 2).

**Table 2:** Color of the peel, outside pulp, pulp and seeds of pawpaw fruits from different cultivars grown in southwest Missouri, 2020.

	**Peel color**			**Outside pulp color**			**Pulp color**			**Seed color**		
**Cultivar**	L*	a*	b*	L*	a*	b*	L*	a*	b*	L*	a*	b*
10–35	39.9 ± 4.0^ab^	3.3 ± 2.9^bcd^	24.1 ± 4.4^a^	44.3 ± 6.8^b^	13.2 ± 3.6^bc^	33.3 ± 8.2^cd^	63.2 ± 6.1^cd^	7.8 ± 4.7^cd^	39.1 ± 4.9^d^	45.8 ± 7.3^a^	1.7 ± 0.8^d^	−0.6 ± 1.5^b^
Overleese	39.3 ± 3.5^abc^	1.5 ± 4.9^d^	19.1 ± 3.2^bc^	50.8 ± 3.9^a^	10.3 ± 2.0^c^	35.7 ± 7.2^bc^	67.9 ± 2.9^bc^	9.4 ± 1.8^bc^	44.7 ± 1.9^abc^	41.0 ± 8.6^abc^	1.6 ± 0.5^d^	−1.0 ± 0.9^b^
PA Golden	38.1 ± 2.7^bc^	2.5 ± 3.7^cd^	16.2 ± 3.8^cd^	51.1 ± 7.3^a^	10.7 ± 3.6^c^	34.0 ± 5.3^bcd^	64.3 ± 8.3^c^	8.9 ± 2.0^bc^	30.3 ± 11.9^e^	38.8 ± 7.2^bcd^	2.8 ± 1.0^ab^	5.2 ± 9.9^a^
Shenandoah	37.9 ± 3.3^bc^	8.4 ± 3.0^a^	20.0 ± 4.9^bc^	53.2 ± 4.3^a^	10.4 ± 1.5^c^	38.0 ± 4.0^abc^	72.1 ± 1.9^ab^	7.6 ± 1.6^cd^	45.5 ± 3.2^a^	43.0 ± 3.9^ab^	2.7 ± 0.9^abc^	1.1 ± 2.4^b^
Sunflower	43.9 ± 3.6^a^	5.6 ± 7.5^abc^	24.6 ± 6.2^a^	53.2 ± 4.5^a^	15.5 ± 4.3^b^	42.6 ± 7.1^a^	74.3 ± 3.2^a^	6.9 ± 2.0^d^	44.8 ± 3.8^ab^	39.6 ± 6.7^bcd^	2.0 ± 0.7^cd^	−0.1 ± 1.5^b^
Susquehanna	39.8 ± 12.0^b^	5.8 ± 3.5^ab^	13.0 ± 7.2^d^	50.4 ± 7.6^a^	20.4 ± 6.0^a^	39.4 ± 12.5^ab^	62.7 ± 6.5^cd^	13.8 ± 2.3^a^	40.2 ± 6.5^bcd^	42.4 ± 5.4^ab^	2.2 ± 1.9^bcd^	0.5 ± 4.5^b^
Wells	34.6 ± 5.1^c^	3.6 ± 3.7^bcd^	14.3 ± 5.4^d^	43.9 ± 5.3^b^	11.8 ± 4.4^c^	28.7 ± 5.1^d^	59.3 ± 8.9^d^	10.1 ± 2.1^b^	33.1 ± 7.3^e^	35.7 ± 3.7^d^	1.9 ± 0.5^d^	0.7 ± 1.7^b^
Wilson	41.8 ± 3.9^ab^	6.6 ± 3.8^ab^	22.4 ± 5.2^ab^	52.4 ± 4.2^a^	9.3 ± 3.1^c^	36.9 ± 6.1^abcd^	69.9 ± 2.2^ab^	9.3 ± 2.6^bc^	38.7 ± 2.9^cd^	35.3 ± 3.2^cd^	3.3 ± 1.1^a^	2.8 ± 2.1^ab^
Control (Mango Fruit)	53.5 ± 6.8	9.7 ± 10.8	35.2 ± 9.0	75.2 ± 2.3	10.3 ± 1.9	71.0 ± 3.6	70.7 ± 2.6	13.3 ± 2.7	71.2 ± 2.9	70.5 ± 2.3	6.2 ± 1.5	51.5 ± 3.9

L*high values indicate light colored, low values indicate dark colored; a^*^high values indicate redness, low values indicate greenness; b^*^high values indicate yellowness, low values indicate blueness.

Values with the same superscripts are statistically similar at p < 0.05.

The color of the pulp layer just beneath the peels (outer pulp) was measured separately from the color of the pulp because the outer pulp was observed to brown more rapidly than the (inner) pulp. This was observed in the lightness values obtained; the outside pulp lightness values (Table 1) for all the cultivars were lower than the lightness values of the pulp indicating the outer pulp was darker than the (inner) pulp (Table 1). Further, the results show that the outside pulp lightness for fruits of the Shenandoah, Sunflower, Wilson, PA Golden, Overleese, and Susquehanna cultivars were not statistically different (p<0.05) (Table 2). However, the lightness of these cultivars was significantly different from the lightness of the outside pulp of fruits of the 10–35 and Wells cultivars (*p* < 0.0001). Also, from the data obtained, the pulp of all the pawpaw cultivars studied recorded higher yellowness and lower redness (Table 2) whereas the outside pulp recorded lower yellowness and higher redness.

The seeds in the Wilson cultivar fruits were the darkest with an average L^*^ value of 35.3 ± 3.2, whereas seeds in the 10–35 cultivar fruits had a relatively lighter color with an average L value of 45.8 ± 7.3 (Table 2).

### Physicochemical and thermal properties of pawpaw pulp

**Figure 4:**
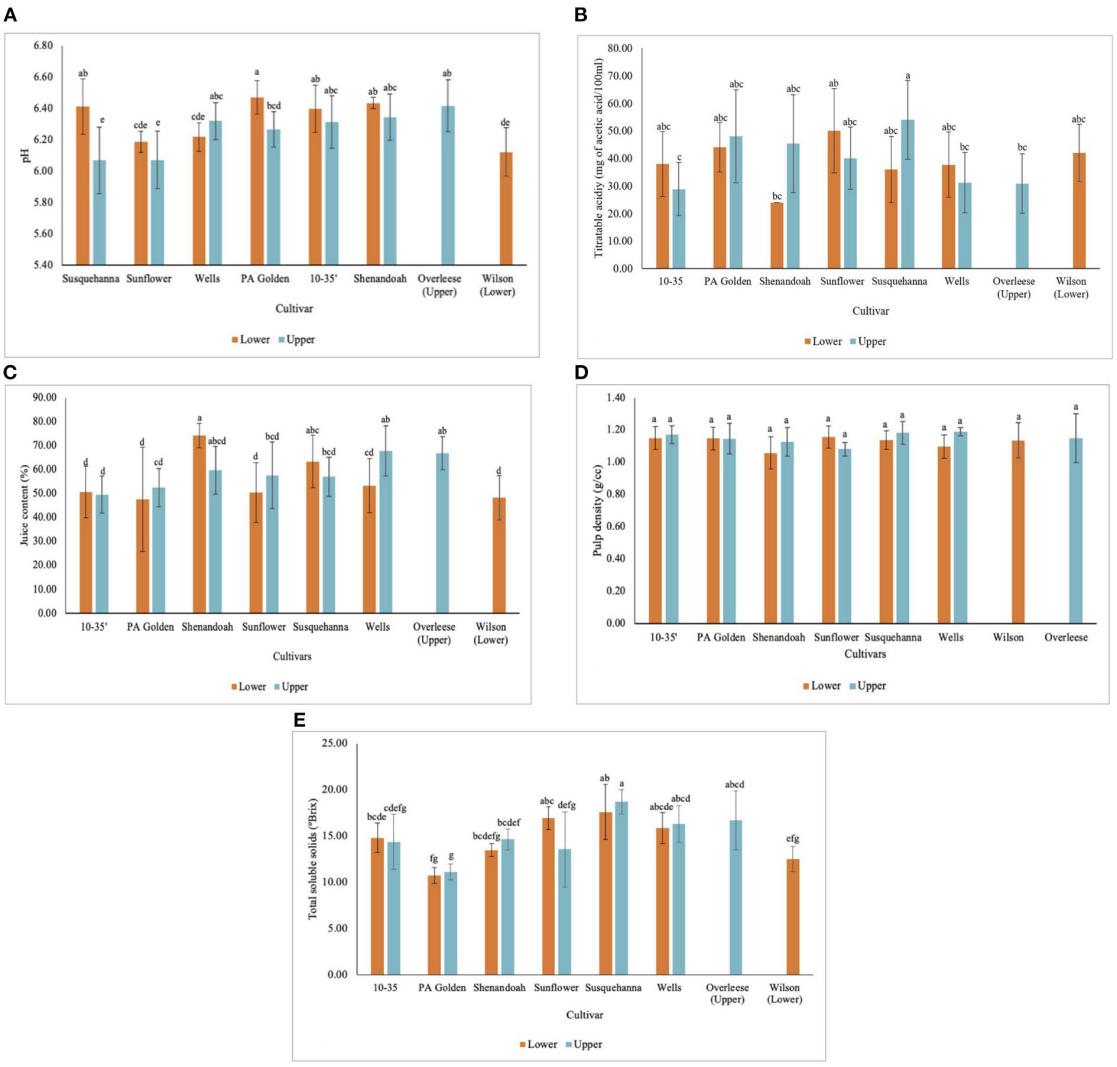
Physicochemical properties of pawpaw fruits from different cultivars in the Lower and Upper orchards showing **(A)** pH, **(B)** titratable acidity, **(C)** juice content, **(D)** pulp density, and **(E)** total soluble solids concentration.

The pH of the fruits from all the cultivars and sites ranged between 6.07 ± 0.21 and 6.47 ± 0.11 (Figure 4A). Overleese fruits recorded the highest pH (6.42 ± 0.17), and the Susquehanna and Sunflower fruits both from the Upper orchard had the lowest pH (6.07 ± 0.21 and 6.07 ± 0.18 respectively) (Table 3 and Figure 4A). While the titratable acidity of the pawpaw cultivars was statistically similar at *p* < 0.05 (Figure 4B), the pH values for the fruits among the different cultivars and the sites were statistically different (*p* < 0.0001).

**Table 3:** Physicochemical properties of pawpaw fruits from different cultivars grown in southwest Missouri, 2020.

**Cultivar**	**Pulp density (g/cm^3^)**	**Juice content (%)**	**pH**	**Total soluble solids (°Brix)**	**Titratable acidity (mg of acetic acid/100ml)**
10–35	1.16 ± 0.06^a^	50.1 ± 9.4^c^	6.36 ± 0.16^abc^	14.64 ± 2.32^bc^	33.82 ± 11.80^a^
Overleese	1.15 ± 0.15^a^	66.8 ± 7.0^a^	6.42 ± 0.17^a^	16.71 ± 3.17^ab^	30.86 ± 10.84^a^
PA Golden	1.15 ± 0.09^a^	50.4 ± 15.7^c^	6.35 ± 0.15^abc^	11.00 ± 0.87^d^	46.67 ± 14.91^a^
Shenandoah	1.10 ± 0.10^a^	64.5 ± 11.0^a^	6.38 ± 0.13^ab^	14.38 ± 1.16^bc^	40.00 ± 17.89^a^
Sunflower	1.12 ± 0.07^a^	54.0 ± 13.7^bc^	6.13 ± 0.15^d^	15.27 ± 3.45^b^	45.00 ± 14.39^a^
Susquehanna	1.16 ± 0.07^a^	60.2 ± 10.2^ab^	6.24 ± 0.26^cd^	18.17 ± 2.38^a^	45.00 ± 15.97^a^
Wells	1.14 ± 0.07^a^	60.9 ± 13.0^ab^	6.27 ± 0.12^bc^	16.14 ± 1.88^ab^	33.88 ± 11.81^a^
Wilson	1.14 ± 0.11^a^	48.2 ± 9.3^c^	6.12 ± 0.15^d^	12.54 ± 1.36^cd^	42.00 ± 10.39^a^
Control (Mango Fruit)	1.15 ± 0.04	73.8 ± 3.2	4.40 ± 0.29	12.78 ± 1.40	255.11 ± 113.89^*^

Values with the same superscripts are statistically similar at p < 0.05.

* Titratable acidity of mango was calculated as milligrams of citric acid/100 ml.

The juice content of the pawpaw fruits varied with statistical significance at *p* < 0.0001. The juice content of the fruits ranged between 47.7 ± 21.8% and 74.2 ± 5.1% for fruits of different cultivars and sites (Figure 4C). On average, Overleese fruits recorded the highest juice content (66.8 ± 7.0%). The same method was used to determine the juice content in fresh mango fruits in this study and it was found that the juice content in the pawpaw cultivars was lower than the juice content in mangoes (73.8 ± 3.2%) as shown in Table 3.

The pulp density of the cultivars studied ranged between 1.06 ± 0.10 g/cm^3^ and 1.19 ± 0.03 g/cm^3^ (Figure 4D). However, there were no significant differences among the cultivars and the sites. Also, the pulp density of the pawpaw fruits was similar to the pulp density of the mango fruits examined (1.15 ± 0.04 g/cm^3^).

The data obtained show that Susquehanna fruits from both orchards recorded the highest TSS concentration (Figure 4E) with an average of 14.38 ± 1.16 °Brix (Table 3). PA Golden fruits had the lowest TSS. The 10–35, Shenandoah and Wilson cultivar fruits had similar TSS concentrations (Table 3).

**Figure 5:**
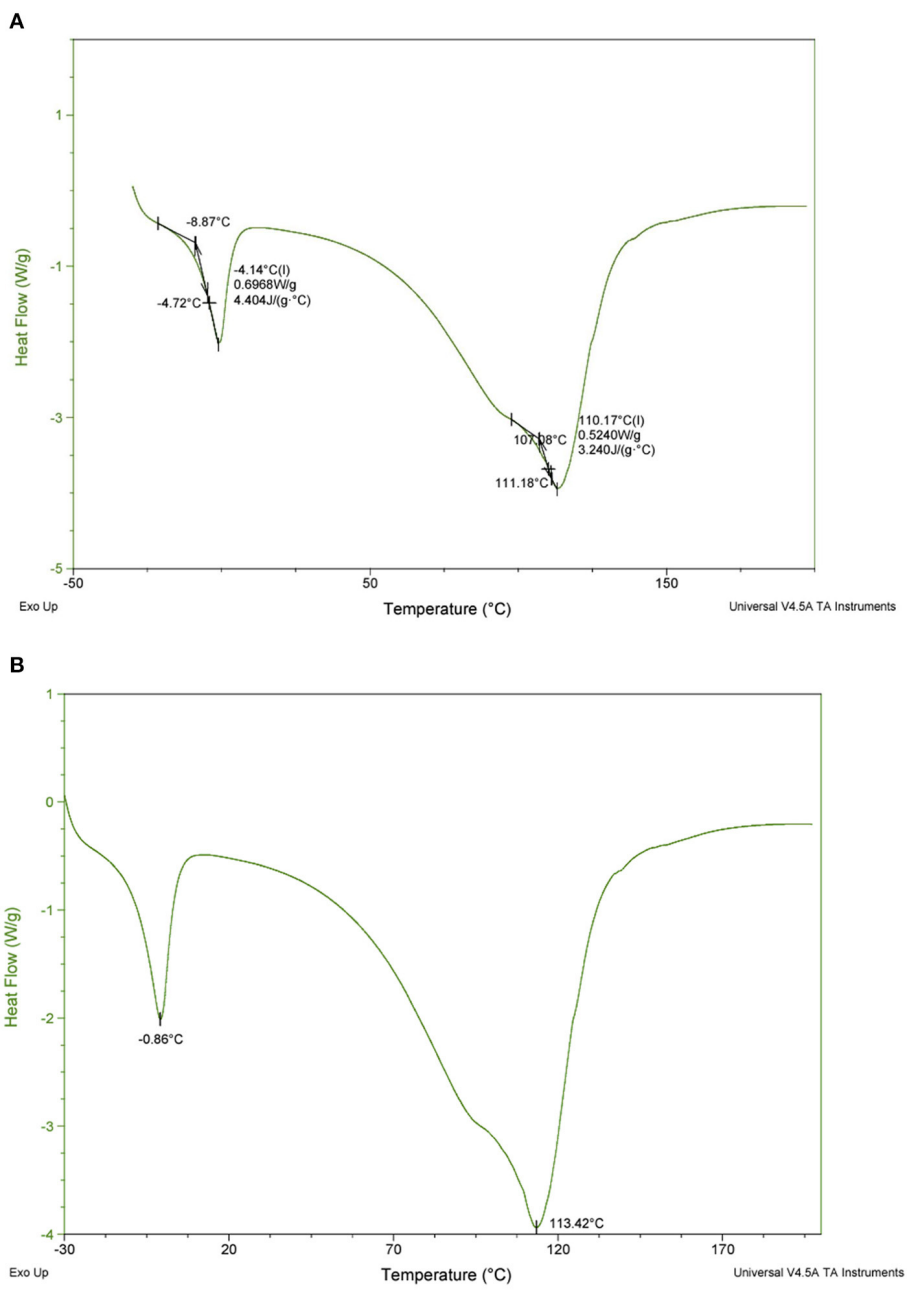
DSC thermogram of pawpaw pulp showing **(A)** glass transition temperature and specific heat capacity, and **(B)** melting and thermal degradation temperatures.

The DSC data obtained show that the glass transition of pawpaw pulp occurs at −8.87°C accompanied by a change in specific heat capacity of 4.404 kJ kg^−1^ K^−1^ (Figure 5A). The peak temperature of ice melting in frozen pawpaw pulp occurs at −0.86°C and the thermal decomposition of the pulp occurred at 113.42°C (Figure 5B).

### Microstructure of pawpaw pulp

**Figure 6:**
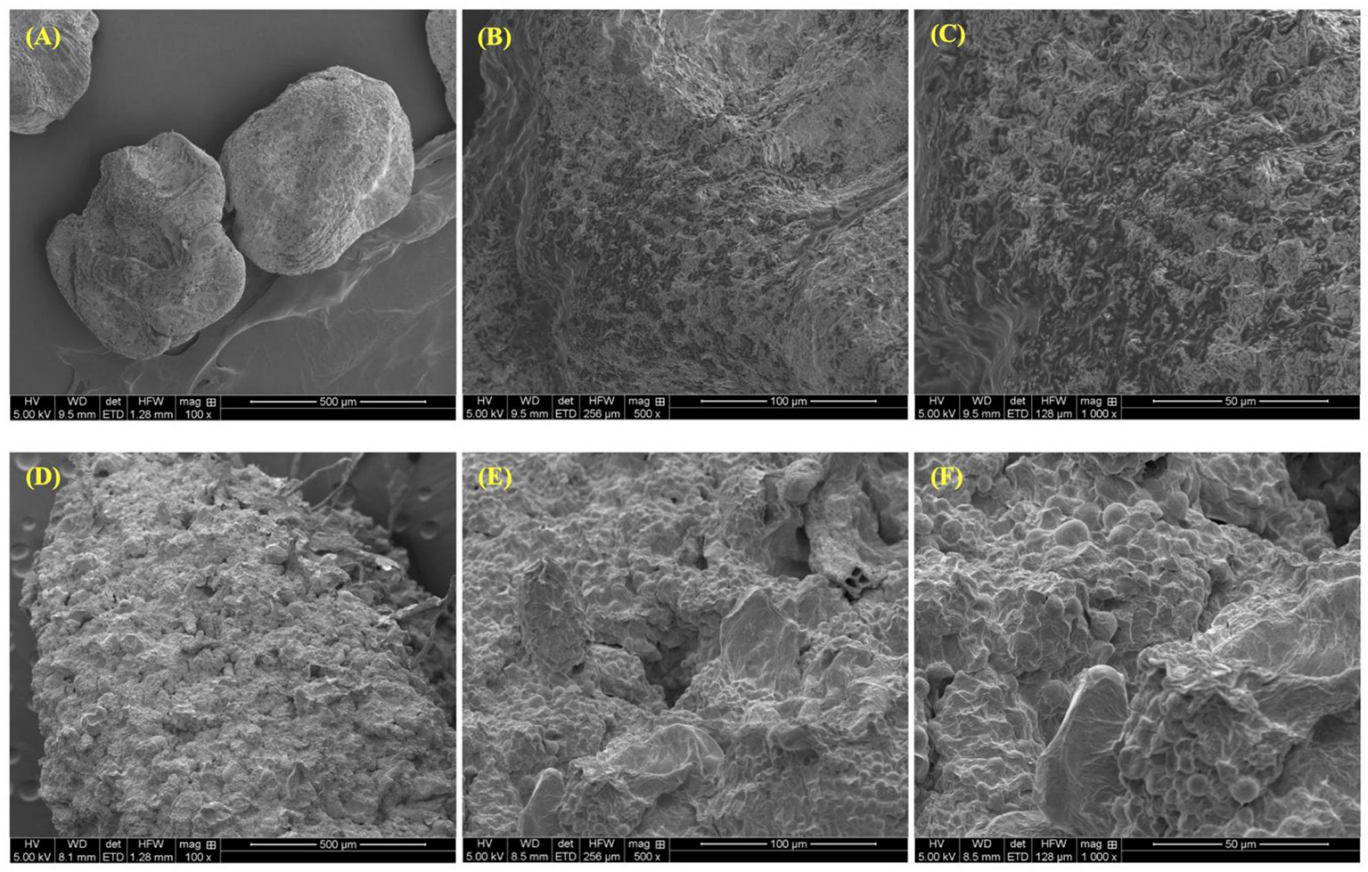
Scanning electron microscope images showing pawpaw pulp close to the seeds **(A–C)** and pawpaw pulp further from the seeds **(D–F)** at magnifications of 100x, 500x, and 1000x.

The SEM (scanning electron microscope) images show a clear distinction in the microstructure of the pulp close to the seed and the pulp further from the seeds (Figure 6). The pulp closer to the seeds showed a smoother surface with no fibers (Figures 6A–C), whereas the pulp further from the seeds showed a more irregular surface with fibers (Figures 6D,E).

## Discussion

### Size and morphological characteristics of pawpaw

Fruit size is an important characteristic that is needed in the selection and design of appropriate processing equipment and is also important in cultivar development. Factors that are known to influence fruit size include genetics, crop load on trees, tree age and vigor, soil nutrients, water supply, pollination, and environmental factors like temperature, humidity, pests, and disease. For pawpaw, studies show that fruit size is affected by cultivar (25), and this was observed in the variations in the fruit lengths and widths. The length and width of the fruits studied were all within the range reported for fresh fruits by (26). This indicates that the fruit length and width were not affected by the freeze-thaw cycle. FSI is an indicator of fruit shape influenced by the genetic makeup of the fruit. FSI >1 indicates an elongated fruit, FSI equal to 1 indicates a round fruit, and FSI < 1 indicates a squat fruit (18). The FSI data show that all the fruits analyzed were elongated, but the fruits of the 10–35 cultivar were the most elongated.

Weight is often used as a quality indicator for fruits and many other agricultural products. Generally, fruits that weigh more have a higher pulp weight, which results in more efficient processing. However, it is important to consider other characteristics of the pulp aside its weight (such as the pH, titratable acidity, total soluble solids content among others) to achieve a desirable quality product when processing the fruit. Hence, if processors choose Susquehanna fruits based on their high pulp weight per fruit, it would also be necessary to carefully consider how the properties of the Susquehanna pulp could influence the quality characteristics of the product they intend to make from the fruit. The weights of the Susquehanna, Wells, and Wilson, fruits were higher than the average weights for the same cultivars as reported by Pomper et al. (6). On the other hand, the weight of the Overleese fruits was lower than the average reported by Pomper et al. (6), but Sunflower fruits had similar weights compared to the average reported by Lolletti et al. (11). It is unclear if freezing had any effect on the weights of the fruits studied. The differences in the experimental data and reported data may have resulted from the differences in the soil quality and environmental conditions of the Lower and Upper orchards as compared to the sites from which the fruits for reported data were obtained. Fruit volume is an important quality index that is used to predict the best time to harvest fruits (27) and to determine fruit expansion rate (19). The volume of the fruits followed a similar trend as the weight of the fruits; the heavier fruits had high volumes.

Peel thickness provides an understanding of how easily fresh fruits may bruise during handling and transportation (28). Additionally, the peel thickness can provide some guidance in the selection and/or design of appropriate industrial peelers to allow for efficient peeling of the fruit before pulp extraction and processing. Peel thickness is influenced by the maturity of fruits; peels of more matured fruits are thinner compared to peels of less matured fruits. Further, peel thickness is an important parameter associated with fruit quality (29) and because the fruits used in this study were frozen and thawed prior to analyses, it is likely that the peel thickness of the fruits were affected by the freeze-thaw cycle prior to measurements. Ripe pawpaw fruits are delicate and easily damaged, hence breeding or producing fruits with thicker peels should significantly reduce bruising and losses that may occur during post-harvest transportation and handling. Studies have shown that fruits with thicker peels are less susceptible to bruising as observed in fruits like pomegranates (28, 30) and banana (31). Hence, for fresh pawpaw marketing, fruits of the Wells cultivar may be preferred as they may not bruise as easily during handling compared to fruits of the other cultivars. Generally, in industrial fruit pulp extraction and processing, various peeling technologies are used. These peeling technologies include mechanical peelers which may be calibrated to peel fruits with peel thickness ranging between 1 and 4 mm (32, 33). However, since the pawpaw fruits have thinner peels (4–13 times thinner than those of mangoes), industrial peelers for other fruits of similar shape and size (like mangoes) may be recalibrated for peeling of pawpaw fruits during industrial processing of pawpaw fruits.

### Pawpaw color

Unlike other fruits where the peel color can be used to determine ripeness, peel color alone is not a good indicator of ripeness in pawpaw fruits (13). Browning of the peel and pulp results in lower lightness (L^*^) values (34), hence, the lightness and darkness of the pawpaw fruit peels could have been a result of the degree of browning that might have occurred in the peels possibly due to the chill injury that had occurred in the peels of the fruits during the freezing of the fruits. The fruits of the Wells cultivar have peels that had the darkest peel color compared to the fruits of the other cultivars. Also, the Sunflower and Wilson fruits peel studied were darker, redder, and less yellow than the Sunflower and Wilson fruit peels studied by Lolletti et al. (11), confirming the effect of freezing on the peel color of the fruits. The lightness of the peels of the fruits was quite consistent for fruits of the same cultivar from the different orchards, indicating that the differences in soil and environmental conditions did not have much effect on the fruit colors even though the freeze-thaw cycle could have affected the data obtained. Hence, to get a better understanding of the effect of soil and environmental conditions on pawpaw fruits, further studies with fresh fruits would need to be conducted.

The data obtained shows that the outer pulp layer had a higher degree of redness compared to the pulp which may have resulted from a higher polyphenol oxidase (PPO) activity in the outer pulp layer. A high PPO activity results in more browning (35). Based on this, during the processing of the fruits, high-pressure processing can be employed to effectively inhibit the activity of PPO in pawpaw pulp without affecting the sensory attributes of the pulp (36). Alternatively, it may be helpful to blanch the fruits after peeling to stop enzymatic browning in the outer pulp layer and the pulp itself. Infrared or microwave blanching treatment can be given to fruits for a limited period to inhibit the activity of enzymes that cause browning and preserve the natural color of the food (37). Maintaining the creamy white/yellow/orange color of pawpaw pulp during processing is a critical step because when the pulp browns, it may no longer be appealing to consumers. Enzymatic browning causes a decline in favorable sensory attributes during processing and storage making it the second major cause of quality loss in fruits and vegetables (38, 39).

The pulp of the Shenandoah fruits studied had a lighter color but a redder color and a more yellow color compared to those reported by Zhang et al. (36). Further, the pulp from the Overleese fruits had a darker color but similar redness and yellowness compared to the Overleese pulp data reported by Brannan et al. (1). This data suggests that freeze-thaw cycles coupled with variations in soil and environmental conditions can affect the color of pawpaw pulp in different ways depending on the fruit cultivar. Analysis of pawpaw pulp kept in frozen storage shows that over time, the frozen pulp is darker and more yellow compared to the fresh pulp (14) as observed in the data obtained for the Shenandoah and the Overleese fruits studied.

### Physicochemical and thermal properties of pawpaw pulp

In this study, the acidity of pawpaw fruits was determined by measuring both the pH and titratable acidity of the pawpaw pulp. A study by Nam et al. (3) shows that pawpaw fruit contains acetic, formic, oxalic, malic, and citric acids, with acetic acid being the predominant acid. Freshly harvested pawpaw fruits have a pH of 6.5, however, as ripening progresses, the acidity increases and then decreases to a pH of 5.2 after 8 weeks of cold storage (15). Further, Francino (25) reported that pawpaw fruits less ripened tend to have a higher pH. The pH values obtained in this study are similar to the values obtained by Galli et al. (15) but higher than the pH values obtained for ripe fruits (Davis cultivar) by Donno et al. (26). Nonetheless, the mango fruits tested had a pH of 4.40 ± 0.29, hence, more acidic than the pawpaw fruits. To successfully use pawpaw fruits in food applications such as jams, jellies, and wine, which require high acidity, more acid would need to be added in the pawpaw preparation to achieve a similar acidity and gel formation as in the mango preparation. Also, the low acidity (almost neutral pH) of the pawpaw pulp may be another contributing factor to its rapid browning on exposure to air. Studies have shown that acidifying agents such as ascorbic acid and citric acid can lower pH and inhibit the action of PPO, slowing enzymatic browning in fruits (40). In pawpaw pulp, studies have demonstrated that lowering the pH of the pulp with ascorbic acid has the potential to inhibit significant color changes for up to 45 days of frozen storage (36).

Fruit juice content is an indicator of fruit maturity. Generally, the juice content in fruits increases as the fruit matures and then declines after the fruit has reached full maturity (41). The results obtained suggest that among the cultivars examined, Overleese fruits may be the best for fruit juice applications of the pawpaw fruit. It is also important to note that all the Overleese fruits used in this study were from only the Upper orchard. The percentage juice contents obtained were higher than the values reported for orange, sweet lime, lemon, and grapes by Jamil et al. (22) although pawpaw pulp has a thicker consistency and about the same moisture content. The high juice contents obtained for the pawpaw fruits studied may be a result of changes that occurred in the fruit during thawing before analyses. Also, the differences in the soil conditions of the Lower and Upper orchards did not have a clear effect on the juice contents of the fruits in the Lower and Upper orchards. Despite this, industrial pawpaw juice extraction may require the use of mechanical juice extractors that can handle the fruit's thick consistency. Alternatively, enzymatic treatment may need to be used in pawpaw pulp prior to juice extraction since pulp treatment with enzymes like pectin methyl esterase and polygalacturonase has been shown to ease juice extraction and increase fruit juice yield in various fruits (42).

Fruit density is often used to predict chemical composition such as dry matter, soluble solids, starch content, and physical disorders (43). Also, the density of the fruit can be used to predict the thermophysical properties of the fruit, which will be useful during its cold storage and processing. In a study that assessed the relationship between fruit density and quality characteristics, denser fruits contained more sugar, polyphenols, and volatile compounds (43). Based on the similarities in the pulp densities of pawpaw pulp and mango pulp, the cold storage conditions used for the storage of mango pulp may be applied for the storage of pawpaw pulp, though they may have different thermal diffusivities due to differences in specific heat capacity and thermal conductivity.

The progression of ripening in pawpaw leads to an increase in the total soluble solids (TSS) and the release of flavor volatiles (1). The TSS of all the cultivars studied were lower than the data reported by Lolletti et al. (11) for NC1 and Taylor cultivars but similar to the data reported for the Sunflower cultivar. It is possible that the freeze-thaw cycle could have influenced the TSS of the pawpaw fruit since it has been shown to significantly alter the TSS of some fruits (44). However, the effect of the freeze-thaw cycle on the TSS of the pawpaw fruit is unclear. Further studies need to be conducted to clearly understand the effect of freeze-thaw cycles on the TSS of pawpaw fruits. Studies have shown that TSS concentration has a significant effect on the inactivation of PPO in a high-pressure processing treatment. Enzymes such as polyphenol oxidases and peroxidases in fruits with higher TSS concentrations have some resistance to inactivation in high-pressure processing treatment (36, 45). Hence, in high-pressure processing of pawpaw fruits to inactivate the PPO and other enzymes that cause browning, Susquehanna fruits may require more pressure to achieve the same level of enzyme inactivation as the PA Golden fruits.

The thermal properties of fruit pulp are critical for designing processing operations that involve heating and/or cooling. Also, since high-pressure processing has been suggested to be a suitable technology for extending the shelf life of pawpaw (36), obtaining the thermal properties of the fruit pulp is very important as these parameters are essential for designing the processing operation (46). The melting temperature of the ice in frozen pawpaw pulp obtained in this study may help improve the storage and processing conditions of pawpaw to make the fruit easier to commercialize. In future studies, investigating other thermal properties like the thermal conductivity, specific heat capacity and enthalpy at different temperatures would be helpful in better understanding the heating and cooling behaviors of pawpaw pulp.

### Effect of freezing on pawpaw fruits and microstructural properties

In our study, we observed that parts of the fruit pulp had a rubbery texture, while other parts had a fibrous texture. To confirm our observations, SEM analysis of pulp samples taken closer to the seeds of the fruit showed a smooth, almost regular surface with no fibers. Meanwhile, SEM analysis of pulp samples taken further from the seeds revealed that a portion of the pulp was fibrous with polygonal and irregular structures on the surface. While this could have been as a result of chill injury leading to changes in the microstructure of the pulp, these structural differences may likely be due to compositional differences between those two parts of the fruit. Studies have shown that portions of fruits with high concentrations of starch or pectin tend to exhibit similar polygonal and irregular surface morphologies as observed in the SEM images of the pawpaw pulp samples taken further from the seeds (47, 48).

Storage temperature has been demonstrated to affect the quality characteristics of fruits. Obenland et al. (49) demonstrated that mandarins stored at lower temperatures had a reduced flavor quality, and high soluble solids concentration to titratable acidity ratio with an increased soluble solids concentration. It is possible that the freezing temperature at which pawpaw fruits were stored before the analyses could have affected the soluble solids, acidity, and other quality characteristics. Further, visual observations made during the experiments show that the pawpaw fruits had undergone chill injury during the frozen storage period. Galli et al. (50) indicated that the loss of antioxidant protective systems (a system that involves enzymes and antioxidants such as reduced glutathione and total ascorbate) during prolonged low-temperature storage significantly promotes chill injury in pawpaw fruits. Therefore, it is critical to optimize the frozen storage of pawpaw fruits considering the volumetric enthalpy changes (Δ*H*_1_ and Δ*H*_2_), Biot's number (*N*_*Bi*_), initial temperature, final center temperature (*T*_*a*_), and mean freezing temperature (*T*_*fm*_) as shown in Pham's equations below, to adequately store pawpaw fruits, where *t* is the freezing time, *d* is a characteristic dimension (radius), *h* is the convective heat transfer coefficient and *E*_*f*_ is the shape factor (51).


(1) $$\begin{array}{l} T_{fm}=1.8+0.263T_{c}+0.105T_{a} \end{array}$$

(2) $$\begin{array}{l} t=\frac{d}{E_{f}h}\left[\frac{\Delta H_{1}}{\Delta T_{1}}+\frac{\Delta H_{2}}{\Delta T_{2}}\right]\left(1+\frac{N_{Bi}}{2}\right) \end{array}$$

Using these equations, a better cold storage system can be designed or adapted for the storage of pawpaw fruits to help retain quality attributes. To have a better understanding of the fruit devoid of the influence of chill injury, there is a need for further studies on fresh pawpaw samples.

## Conclusion

The findings presented in this paper show that there are variations in the physical properties of frozen fruits from the eight pawpaw cultivars studied. Among the cultivars studied, Susquehanna fruits had the highest total fruit weight, pulp weight, volume, and total soluble solids concentration. This could potentially make Susquehanna fruits the preferred cultivar for pawpaw pulp processing. However, it is important to consider all other quality characteristics of the fruits when processing to produce a desirable high-quality product. Further, fruits of the Susquehanna cultivar had the highest fruit length, fruit width, and fruit thickness; nonetheless, these dimensions were found to be similar to mangoes, suggesting the fruit peelers designed for other fruits with similar shape and size like mangoes may be suitable for peeling pawpaw for industrial processing. Due to the pawpaw fruits' thinner peels, such fruit peelers may need to be optimized to reduce pulp wastes during pawpaw peeling. It is likely the peel thickness, peel color, and pulp color of the fruits were influenced by the freeze-thaw cycle as well as the soil and environmental variations but to different extents for the different cultivars and orchards. Fruits of the Wells cultivar may be less susceptible to bruising since they had the thickest peels of the cultivars studied. This might make them more suitable for the fresh pawpaw markets. Also, the fruits of the Sunflower cultivar had the highest peel yellowness and peel lightness. These color indicators may be helpful for farmers who plan to grow pawpaw fruits for the fresh fruit markets to be sold in grocery shops; nonetheless, due to the fruit's rapid browning, the appropriate storage mechanisms must be applied to make high-quality fresh fruits available to consumers. Overall, since pawpaw pulp has an almost neutral pH, it would be necessary to acidify the pulp or use high-pressure processing to inhibit the enzymatic browning that occurs in the pulp during storage. Juice extraction from pawpaw fruits may be more feasible with Overleese fruits than fruits from other cultivars. Potentially, the use of enzymatic treatments could ease juice extraction from all the pawpaw cultivars, and the pulp could also be used in other food applications including jams and jellies. These findings set the stage for further studies on fresh pawpaw fruits since this study was carried out with frozen samples. This will provide further understanding to develop effective postharvest loss prevention strategies and extend the shelf life of pawpaw fruits. Also, due to the diversity of genetics, there is no perfect fruit suitable for all purposes. Hence, it might be necessary to develop cultivars for specific purposes, such as cultivars for fresh fruit marketing and cultivars for fruit processing, to further ease the commercialization of the fruit.

## Data availability statement

The original contributions presented in the study are included in the article/Supplementary material, further inquiries can be directed to the corresponding author.

## Author contributions

BA: investigation, data curation, formal analysis, writing–original draft, writing–review and editing, and visualization. BC: investigation and formal analysis. AT: resources, funding acquisition, and writing–review and editing. C-HL: writing–review and editing. ZC, PB, and MG: resources and writing–review and editing. KK: conceptualization, methodology, funding acquisition, investigation, writing–review and editing, and supervision. All authors contributed to the article and approved the submitted version.

## Funding

This work is supported by the University of Missouri Center for Agroforestry and the USDA/ARS Dale Bumpers Small Farm Research Center, Agreement number 58-6020-0-007 from the USDA Agricultural Research Service, USDA Hatch Funds - FEAST Lab (MO-HAFE0003) and a USDA Specialty Crop Block Grant through the Missouri Department of Agriculture.

## Conflict of interest

The authors declare that the research was conducted in the absence of any commercial or financial relationships that could be construed as a potential conflict of interest.

## Publisher's note

All claims expressed in this article are solely those of the authors and do not necessarily represent those of their affiliated organizations, or those of the publisher, the editors and the reviewers. Any product that may be evaluated in this article, or claim that may be made by its manufacturer, is not guaranteed or endorsed by the publisher.
