# Supplementary figures and images for: Physical characterization of frozen fruits from eight cultivars of the North American pawpaw (Asimina triloba)
Source: Front Nutr. 2022 Oct 18;9:936192. doi: 10.3389/fnut.2022.936192 (PMC9622945; doi:10.3389/fnut.2022.936192)

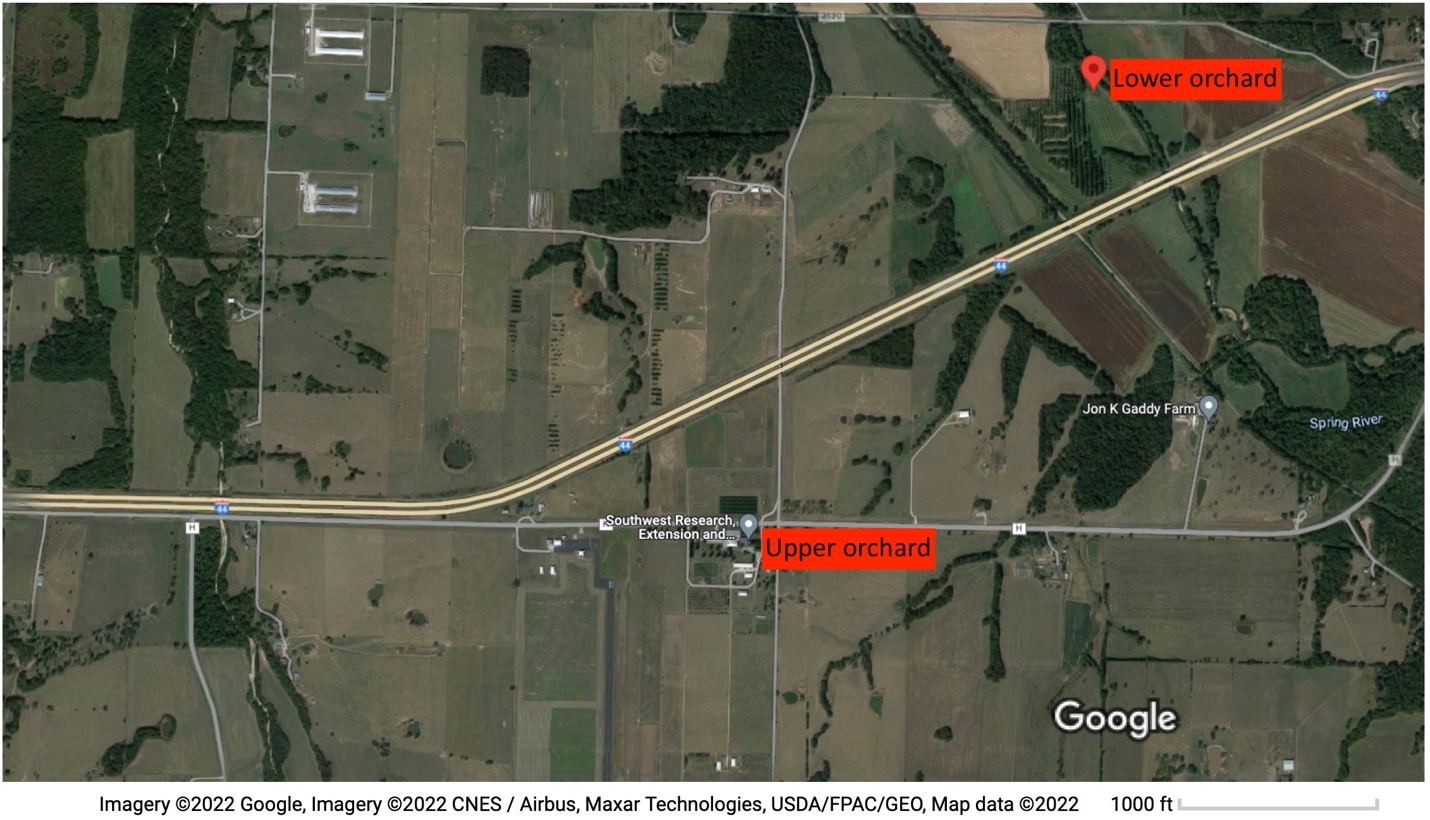


Supplementary Figure 1: Map of locations where pawpaw fruits were harvested

Supplement: Supplementary file 1 [file Data_Sheet_1.docx]
